# Supplementary material for: Genomic and Glycolytic Entropy Are Reliable Radiogenomic Heterogeneity Biomarkers for Non-Small Cell Lung Cancer
Source: Int J Mol Sci. 2023 Feb 16;24(4):3988. doi: 10.3390/ijms24043988 (PMC9959107; doi:10.3390/ijms24043988)
Supplement: Supplementary file 1 [file ijms-24-03988-s001.zip › ijms-2159402-supplementary.pdf]

Supplementary Materials

Genomic and glycolytic entropy are reliable radiogenomic heterogeneity biomarkers for non-small cell lung cancer

Yu-Hung Chen [1,2,3], Kun-Han Lue [3], Chih-Bin Lin [4], Kuang-Chi Chen [5], Sheng-Chieh Chan [1,2], Sung-Chao Chu [2,6,*], Bee-Song Chang [7], and Yen-Chang Chen [2,8]

[1] Department of Nuclear Medicine, Hualien Tzu Chi Hospital, Buddhist Tzu Chi Medical Foundation, Hualien 97002, Taiwan

[2] School of Medicine, College of Medicine, Tzu Chi University, Hualien 97002, Taiwan

[3] Department of Medical Imaging and Radiological Sciences, Tzu Chi University of Science and Technology, Hualien 97005, Taiwan

[4] Department of Internal Medicine, Hualien Tzu Chi Hospital, Buddhist Tzu Chi Medical Foundation, Hualien 97002, Taiwan

[5] Department of Medical Informatics, Tzu-Chi University, Hualien, Taiwan

[6] Department of Hematology and Oncology, Hualien Tzu Chi Hospital, Buddhist Tzu Chi Medical Foundation, Hualien 97002, Taiwan

[7] Department of Cardiothoracic Surgery, Hualien Tzu Chi Hospital, Buddhist Tzu Chi Medical Foundation, Hualien 97002, Taiwan

[8] Department of Anatomical Pathology, Hualien Tzu Chi Hospital, Buddhist Tzu Chi Medical Foundation, Hualien

* Correspondence: oldguy-chu1129@umail.hinet.net (Sung-Chao Chu); Tel.: +886-3-856-1825 ext 12020 (Sung-Chao Chu).

Table S1. Comparison of genomic features between different smoking, histology, and actionable EGFR mutation status.

Variable	Smoking status (mean ± SD)			Histology (mean ± SD)			EGFR status (mean ± SD)		
	Ever smoker (n = 27)	Never used (n = 19)	*p*	Adenocarcinoma (n = 35)	Squamous cell (n = 11)	*p*	Mutant (n = 16)	Wild type (n = 30)	*p*
TMBc	20.7 ± 11.06	15.7 ± 2.59	0.033*	18.1 ± 8.37	20.3 ± 10.74	0.558	15.9 ± 2.94	20.1 ± 10.61	0.051
TMBm	14.0 ± 11.02	9.2 ± 2.62	0.038*	11.5 ± 8.37	13.7 ± 10.55	0.545	9.5 ± 2.94	13.4 ± 10.59	0.71
MATHc	47.8 ± 13.61	46.2 ± 16.10	0.723	47.0 ± 15.01	47.3 ± 13.57	0.958	46.6 ± 16.20	47.4 ± 13.85	0.885
MATHm	53.5 ± 13.38	58.3 ± 12.1	0.211	56.5 ± 12.35	52.1 ± 14.89	0.380	58.6 ± 11.89	53.8 ± 13.39	0.213
Shannon entropy.c	2.8 ± 0.21	2.8 ± 0.11	0.323	2.8 ± 0.13	2.9 ± 0.25	0.159	2.8 ± 0.12	2.8 ± 0.20	0.866
Shannon entropy.m	2.6 ± 0.26	2.6 ± 0.20	0.624	2.6 ± 0.20	2.7 ± 0.32	0.201	2.6 ± 0.19	2.6 ± 0.26	0.671
SUVmax	12.9 ± 7.61	10.6 ± 5.99	0.265	11.3 ± 5.81	14.1 ± 9.99	0.397	11.3 ± 5.89	12.3 ± 7.61	0.654
MTV	38.8 ± 69.3	16.3 ± 18.62	0.119	30.8 ± 60.42	25.3 ± 35.38	0.716	14.7 ± 16.06	37.3 ± 66.4	0.086
TLG	396.7 ± 813.66	141.1 ± 220.84	0.131	304.8 ± 713.17	247.8 ± 387.23	0.736	133.8 ± 203.35	375.0 ± 778.86	0.119
Entropy	4.3 ± 0.83	4.1 ± 0.87	0.389	4.2 ± 0.80	4.3 ± 1.00	0.722	4.2 ± 0.72	4.2 ± 0.91	0.912

EGFR, epidermal growth factor receptor; SD, standard deviation; TMB, tumor mutation burden; MATH, mutant-allele tumor heterogeneity; SUV, standardized uptake value; MTV, metabolic tumor volume; TLG, total lesion glycolysis. *Statistically significant.

Figure S1. The effect of PAF cutoff changes on the calculation of genomic heterogeneity.

The genomic heterogeneity features in a patient with a single-peaked MAF distribution pattern showed a minor shift when different PAF cutoffs were applied (a and b). In the bimodally distributed MAF pattern (c and d), the MATH shifts more apparently after PAF cutoffs change, whereas Shannon entropy showed only minor changes.

PAF, population allele frequency; MAF, mutant allele frequency; MAD, median absolute deviation; MATHc and MATHm, mutant-allele tumor heterogeneity based on PAF cutoffs of 1% and 0.01%; Shannon entropy.c and Shannon entropy.m, Shannon entropy based on PAF cutoffs of 1% and 0.01%.

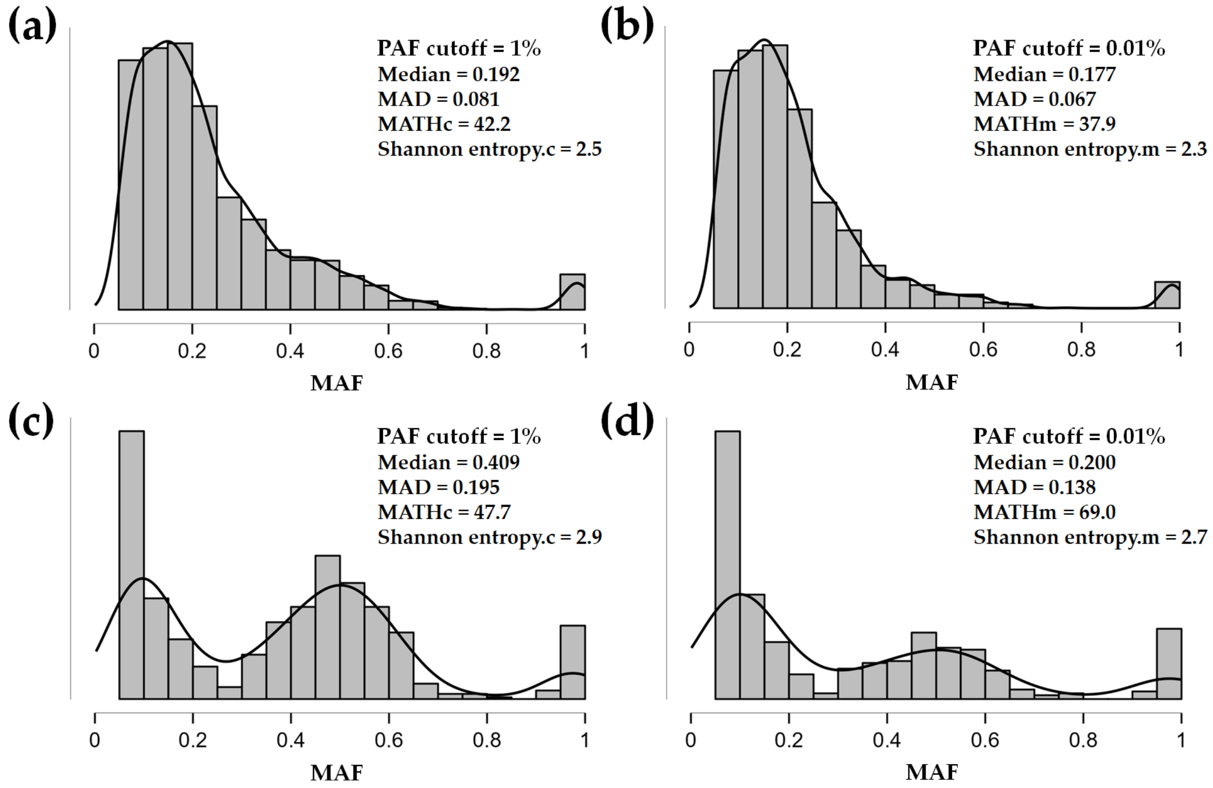

Figure S2. The ICC of ^{18}F-FDG PET-derived radiomic features between standard (256 × 256) and reduced (128 × 128) image matrix sizes. Features were extracted on Pyradiomics 2.2.0 (Harvard Medical School, Boston, MA, USA) using a fixed bin size of 0.25 SUV.

^{18}F-FDG PET, ^{18}F-Fluorodeoxyglucose positron emission tomography; ICC, intraclass correlation coefficient; SUV, standardized uptake value; MTV, metabolic tumor volume; TLG, total lesion glycolysis; GLCM, Gray Level Co-occurrence Matrix; NGTDM, Neighbouring Gray Tone Difference Matrix; GLRLM, Gray Level Run Length Matrix; Gray Level Size Zone Matrix.

	ICC		
First-order features	All	MTV<10	MTV>10
SUV_{max}	0.99	0.96	0.992
MTV	0.998	0.98	0.998
TLG	0.995	0.991	0.995
Entropy	0.958	0.894	0.993
Skewness	0.951	0.858	0.985
Energy	0.424	0.482	0.42
Kurtosis	0.907	0.706	0.953
GLCM features	All	MTV<10	MTV>10
Cluster Prominence	0.947	0.892	0.946
Cluster Shade	0.888	0.846	0.886
Cluster Tendency	0.98	0.935	0.98
Correlation	0.883	0.857	0.685
Maximum Correlation Coefficient	0.129	0.583	0.043
Inverse Difference	0.976	0.94	0.991
Inverse Difference Moment	0.969	0.912	0.99
joint Energy	0.38	0.234	0.965
joint Entropy	0.87	0.787	0.92
Contrast	0.826	0.837	0.827
NGTDM features	All	MTV<10	MTV>10
Busyness	0.461	0.628	0.461
Coarseness	0.727	0.691	0.579
Contrast	0.271	0.192	0.759
Complexity	0.964	0.89	0.694

	ICC		
GLRLM features	All	MTV<10	MTV>10
Short Run Emphasis	0.899	0.693	0.934
Long Run Emphasis	0.732	0.625	0.733
Gray Level Non-Uniformity	0.495	0.491	0.498
Run Length Non-Uniformity	0.486	0.485	0.486
Gray Level Variance	0.997	0.957	0.997
Run Variance	0.657	0.588	0.653
Run Entropy	0.956	0.899	0.989
Run Percentage	0.884	0.676	0.914
GLSZM features	All	MTV<10	MTV>10
Small Area Emphasis	0.795	0.774	0.865
Large Area Emphasis	0.091	0.016	0.091
Gray Level Non-Uniformity	0.606	0.387	0.608
Size Zone Non-Uniformity	0.693	0.624	0.691
Gray Level Variance	0.996	0.927	0.998
Zone Variance	0.092	0.009	0.092
Zone Entropy	0.926	0.863	0.923
Zone Percentage	0.913	0.868	0.949
